# The Impact of HIV Co-Infection on the Genomic Response to Sepsis

**DOI:** 10.1371/journal.pone.0148955

**Published:** 2016-02-12

**Authors:** Michaëla A. M. Huson, Brendon P. Scicluna, Lonneke A. van Vught, Maryse A. Wiewel, Arie J. Hoogendijk, Olaf L. Cremer, Marc J. M. Bonten, Marcus J. Schultz, Marek Franitza, Mohammad R. Toliat, Peter Nürnberg, Martin P. Grobusch, Tom van der Poll

**Affiliations:** 1 Center for Experimental and Molecular Medicine, Academic Medical Center, University of Amsterdam, Amsterdam, The Netherlands; 2 Centre des Recherches Médicales de Lambaréné, Lambaréné, Gabon; 3 Department of Intensive Care Medicine, University Medical Center Utrecht, Utrecht, The Netherlands; 4 Department of Medical Microbiology, University Medical Center Utrecht, Utrecht, The Netherlands; 5 Julius Center for Health Sciences and Primary Care, University Medical Center Utrecht, Utrecht, The Netherlands; 6 Department of Intensive Care, Academic Medical Center, University of Amsterdam, Amsterdam, The Netherlands; 7 Cologne Center for Genomics (CCG), University of Cologne, Cologne, Germany; 8 Cologne Excellence Cluster on Cellular Stress Responses in Aging-Associated Diseases (CECAD), University of Cologne, Cologne, Germany; 9 Center for Molecular Medicine Cologne (CMMC), University of Cologne, Cologne, Germany; 10 Center of Tropical Medicine and Travel Medicine, Academic Medical Center, University of Amsterdam, Amsterdam, The Netherlands; 11 Division of Infectious Diseases, Academic Medical Center, University of Amsterdam, Amsterdam, The Netherlands; Emory University School of Medicine, UNITED STATES

## Abstract

HIV patients have an increased risk to develop sepsis and HIV infection affects several components of the immune system involved in sepsis pathogenesis. We hypothesized that HIV infection might aggrevate the aberrant immune response during sepsis, so we aimed to determine the impact of HIV infection on the genomic host response to sepsis. We compared whole blood leukocyte gene expression profiles among sepsis patients with or without HIV co-infection in the intensive care unit (ICU) and validated our findings in a cohort of patients admitted to the same ICUs in a different time frame. To examine the influence of HIV infection per se, we also determined the expression of genes of interest in a cohort of asymptomatic HIV patients. We identified a predominantly common host response in sepsis patients with or without HIV co-infection. HIV positive sepsis patients in both ICU cohorts showed overexpression of genes involved in granzyme signaling (*GZMA*, *GZMB*), cytotoxic T-cell signaling (*CD8A*, *CD8B*) and T-cell inhibitory signaling (*LAG3*), compared to HIV negative patients. Enhanced expression of *CD8A*, *CD8B* and *LAG3* was also unmasked in asymptomatic HIV patients. Plasma levels of granzymes in sepsis patients were largely below detection limit, without differences according to HIV status. These results demonstrate that sepsis is characterized by a massive common response with few differences between HIV positive and HIV negative sepsis patients. Observed differences in granzyme signaling, cytotoxic T-cell signaling and T-cell inhibitory signaling appear to be changes commonly observed in asymptomatic HIV patients which persist during sepsis.

## Introduction

Sepsis is a major cause of morbidity and mortality in patients with HIV. In Western settings, sepsis accounts for 33–35% of intensive care unit (ICU) admissions in HIV/AIDS patients, and is associated with high mortality [1, 2]. Similarly, in tropical regions, patients with HIV have higher rates of bacterial bloodstream infections [3]. Sepsis is characterized by the presence of an infection combined with a systemic injurious host response [4, 5]. Of interest, HIV infection affects several components of the immune system in similar ways as sepsis; including leukocyte responses, the complement system, and the coagulation system [6]. However, knowledge on the impact of chronic HIV infection on the host response to sepsis is limited. Two previous studies examined the cytokine response in sepsis patients with or without HIV co-infection and found few differences in cytokine levels [7, 8]. On the other hand, in Malawian children with bacterial sepsis, those with HIV co-infection had more profound increases in angiopoietin 2, an angiogenic peptide that increases endothelial activation and is associated with disseminated intravascular coagulation and mortality in sepsis [9]. Furthermore, we demonstrated an additive effect of HIV co-infection on complement activation during sepsis [10]. These studies suggest that HIV co-infection enhances essential aspects of the pro-inflammatory response during sepsis.

Microarray technology has allowed for great advances in genome level understanding of sepsis [11]. Previous studies using whole genome transcriptome profiling reported massive up-regulation of inflammation- and innate immunity-related genes, as well as suppression of adaptive immunity in patients with sepsis and septic shock [11, 12]. We hypothesized that pre-existing HIV infection aggrevates the aberrant immune response during sepsis. To investigate this assumption we compared whole blood leukocyte gene expression profiles in sepsis patients with or without HIV co-infection admitted to the ICU. Subsequently, we validated our findings in patients admitted to the same ICUs during a different timeframe. To examine the influence of HIV infection per se, we also determined the expression of genes of interest in a cohort of asymptomatic HIV patients from an HIV endemic area.

## Methods

### Patients and definitions

Patients were recruited within the framework of the Molecular Diagnosis and Risk Stratification of Sepsis (MARS) project, an observational cohort study in the ICUs of two tertiary teaching hospitals (Academic Medical Center in Amsterdam and University Medical Center Utrecht) in the Netherlands between January 2011 and January 2014 (clinicaltrials.gov identifier NCT01905033). The Medical Ethical Committees of both study centers approved an opt-out consent method. Participants were notified of the study in writing by a brochure with an opt-out card that could be completed by the patient or by his or her legal representative in case of unwillingness to participate [13]. Dedicated and trained physicians prospectively collected demographics, ICU admission characteristics, daily physiological measurements, severity scores (including Sequential Organ Failure Assessment (SOFA) scores) [14]and culture results. Central nervous system (CNS) scores were excluded from our analysis of SOFA scores since many patients were sedated. Organ failure was defined by a score of 3 or greater on the SOFA score, or a score of 1 or more for cardiovascular failure [15]. Shock was defined by the use of vasopressors (noradrenaline) for hypotension in a dose of 0.1 mcg/kg/min during at least 50% of the ICU day. The plausibility of infection was assessed posthoc, based on all available evidence, and classified on a 4-point scale (none, possible, probable or definite) according to Centers for Disease Control and Prevention [16] and International Sepsis Forum consensus definitions [17], as previously described in detail [13]. Sepsis was defined as the presence of infection (with likelihood other than none) combined with at least one of general, inflammatory, hemodynamic, organ dysfunction or tissue perfusion parameters derived from the 2001 International Sepsis Definitions Conference [18]. Cases were defined by the presence of sepsis and HIV co-infection. For each case, two sepsis patients without HIV infection were selected, matched for age, sex, race, and source of infection. If a perfect match was not available, age was allowed to differ by 5 years and race was allowed to differ. For patients with multiple admissions, only the first admission was selected for analysis. Whole genome transcriptome profiling of blood leukocytes was performed on 20 HIV patients admitted with sepsis, diagnosed within 24 hours after ICU admission, between January 2011 and July 2012, and 40 matched HIV negative patients admitted with sepsis during the same period. Subsequently, gene transcripts differentially expressed in HIV positive and HIV negative patients in the first cohort were determined by reverse transcribed quantitative polymerase chain reaction (qRT-PCR) in a second independent cohort derived from the same two ICUs, comprising 12 HIV positive and 24 matched HIV negative patients admitted with sepsis between July 2012 and January 2014. For HIV infected patients, information on CD4 counts, viral loads and use of combination antiretroviral therapy (cART) was retrospectively collected from patient files. CD4 counts and viral loads measured between 120 days prior to and 30 days after admission were considered representative. If multiple samples were collected in this period, the first sample was included in our analyses. The Municipal Personal Records Database was consulted to determine survival up to one year after ICU admission. To analyze differences between healthy subjects and sepsis patients, blood was also obtained from 42 healthy controls (age 35 (30–63) years, median with interquartile ranges; 57% male) after providing written informed consent.

A third cohort was used to determine the effect of HIV infection per se on the expression of gene transcripts differentially expressed between HIV positive and HIV negative sepsis patients. For this, 60 asymptomatic HIV positive and 33 healthy controls without HIV infection were recruited in the environment of the Albert Schweitzer Hospital (Lambaréné, Gabon) between March 2012 and July 2013, as previously described [10]. The study in Gabon was approved by the scientific review committee of the Centre des Recherches Médicales de Lambaréné (CERMEL). Prior to enrolment, written informed consent was obtained from all participants.

### Whole-blood leukocyte RNA and microarray

For whole-blood leukocyte messenger (m)RNA analyses blood was collected in PAXgene tubes (Becton-Dickinson, Breda, the Netherlands) within 24 hours after ICU admission. Blood was processed for RNA isolation by means of the QIAcube machine (Qiagen, Venlo, the Netherlands) in combination with the Blood mRNA kit (Qiagen) according to manufacturer’s instructions. Total RNA (RNA integrity number > 6.0) was processed and hybridized to the Human Genome U219 96-array plate using the GeneTitan^R^ instrument (Affymetrix, Santa Clara, California, United States) as described by the manufacturer (Affymetrix). Hybridizations and scans were performed at the Cologne Center for Genomics (CCG), Cologne, Germany. Raw data scans (.CEL files) were read into the R language and environment for statistical computing (version 2.15.1; R Foundation for Statistical Computing, Vienna, Austria; http://www.R-project.org/). Pre-processing and quality control were performed by using the *Affy* package (version 1.36.1) [19]. Array data were background corrected by Robust Multi-array Average, quantiles-normalized and summarized by median polish using the *expresso* function (*Affy* package). The resultant 49,386 log2-transformed probe intensities were filtered by means of a 0.5 variance cutoff using the genefilter method to recover 24,646 expressed probes [20]. The occurrence of non-experimental chip effects was evaluated by means of the Surrogate Variable Analysis (R package version 3.4.0) [21] and corrected by the empirical Bayes method ComBat [22]. The non-normalized and normalized MARS gene expression data sets are available at the Gene Expression Omnibus public repository of NCBI under accession number GSE65682.

### Microarray data analysis and bioinformatics

The 24,646 expressed probes were assessed for differential abundance across healthy controls, HIV negative and HIV positive sepsis patients by means of the *limma* R package (version 3.14.4) [23, 24]. Supervised analysis (comparison between pre-defined groups) was performed by moderated *t* statistics. Throughout, Benjamini-Hochberg (BH) multiple comparison adjusted probabilities (BH p<0.05) defined significance. Ingenuity Pathway Analysis (Ingenuity Systems IPA, www.ingenuity.com) was used to evaluate associations with canonical signaling pathways of over- and under-expressed gene patterns. The Ingenuity gene knowledgebase was selected as reference and human species specified. All other IPA parameters were default. Association significance was measured by Fisher’s exact test BH-adjusted p-values (BH p<0.05).

### Quantitative real-time PCR

Total RNA was purified from Paxgene tubes collected within 24 hours after admission to the ICU, or (in the Gabonese cohort) upon enrolment in the study, using the Paxgene Blood RNA kit (Qiagen). RNA was reverse transcribed using oligo (dT) primer and Moloney murine leukemia virus reverse transcriptase (Invitrogen, Breda, the Netherlands) according to supplier’s recommendations. The LightCycler system (LC480, Roche Applied Science, Penzberg, Germany) was used for quantitative reverse-transcription (qRT)-PCR analysis. Results were normalized to the reference gene *HPRT1*. Analysis was performed by linear regression implemented in the LinRegPCR program [25]. Primer sequences were designed so as not to overlap with microarray probes, and are provided in S1 Table.

### Enzyme linked immunosorbent assays

Plasma levels of granzyme A and granzyme B were measured in EDTA anticoagulated plasma obtained for routine care on admission. Left over plasma was processed and stored at -80°C within four hours after blood collection. Protein levels of granzyme A and granzyme B were measured by Enzyme linked immunosorbent assay as previously described [26]. In brief, purified monoclonal antibody GA29 or GB11 were used as capture antibodies and biotinylated GA28 or GB10 monoclonal antibodies were used as detecting antibodies for granzyme A and granzyme B, respectively. The detection limits were 50 pg/ml for granzyme A and 55 pg/ml for granzyme B.

### Statistical analysis

Statistical analysis pertaining to patient demographics, qRT-PCR data and ELISA data were performed using Fisher’s exact tests for comparisons of categorical variables, Mann Whitney U tests or Kruskall-Wallis rank sum tests to assess differences for non-normally distributed continuous variables, and unpaired t-tests or one-way ANOVA tests for normally distributed variables. A Kolmogorov-Smirnov test was applied to determine normality. For correlation analyses, we calculated Spearman rank correlation coefficients (all data for these analyses were non-normally distributed). A p-value <0.05 defined significance in all analyses.

## Results

### Characteristics and outcome of patients in the genomic response cohort

We analysed the whole blood leukocyte transcriptome upon ICU admission for sepsis in 20 HIV positive and 40 matched HIV negative patients (Table 1). As expected, due to matching, sex, age, race and source of infection did not differ between groups. The majority of patients was male (85%), and the median age of the entire cohort was 50 (43–60) years. The most common type of infection was respiratory tract infection (55%). Causative pathogens were largely similar between HIV positive and HIV negative patients, with the exception of *Pneumocystis jirovecii*, which was found exclusively in HIV patients (p = 0.033) (S2 Table). The severity of illness upon ICU admission did not differ between groups, as indicated by SOFA scores and the proportion of patients presenting with organ failure or shock. Likewise, mortality up to one year after ICU admission was similar in HIV positive and HIV negative sepsis patients. Mortality in sepsis patients with HIV co-infection was not related to viral loads or CD4 counts, except for a survival benefit after one year in patients with higher CD4 counts (median 425 (317–530) in survivors compared to 225 (10–340) in non-survivors, p = 0.02).

**Table 1:** Characteristics of Dutch ICU patients in the genomic response cohort and validation cohort.

	Genomic response cohort			Validaton cohort		
	HIV+ n = 20	HIV- n = 40	P	HIV+ n = 12	HIV- n = 24	P
**Demographics**						
Male (%)	17 (85.0)	34 (85.0)	1.0	8 (66.7)	16 (66.7)	1.0
Age [IQR]	48 [44–59]	50 [43–61]	0.86	48 [40–60]	49 [40–60]	0.76
Race: white (%)	12 (60.0)	28 (70.0)	0.56	9 (75.0)	19 (79.2)	0.79
Race: black (%)	7 (35.0)	9 (22.5)	0.36	2 (16.7)	5 (20.8)	1.0
Race: asian (%)	1 (5.0)	2 (5.0)	1.0	1 (8.3)	0 (0.0)	0.33
Race: unknown (%)	0 (1.7)	1 (2.5)	1.0	—	—	—
**Primary source of infection**						
Respiratory tract (%)	11 (55.0)	22 (55.0)	1.0	11 (91.7)	22 (91.7)	1.0
Central nervous system (%)	4^A^ (20.0)	7 (17.5)	1.0	—	—	—
Skin or soft tissue (%)	2 (10.0)	5 (12.5)	1.0	—	—	—
Intra-abdominal (%)	2 (10.0)	5 (12.5)	1.0	—	—	—
Cardiovascular (%)	0 (0.0)	1 (2.5)	1.0	—	—	—
Urinary tract (%)	—	—	—	1 (8.3)	2 (8.3)	1.0
Primary bacteremia (%)	1 (5.0)^B^	0 (0.0)	0.33	—	—	—
Antiretroviral therapy (%)	14 (70.0)	—	—	5 (41.7)	—	—
CD4 count (cells/mm^3^) [IQR]	290 [37–378]			45 [13–124]		
Viral load (copies/ml) [IQR]	100 [<50–20180]			4.22x10^4^ [<50–39.56x10^4^]		
SOFA score^C^ [IQR]	9 [5–14]	7 [4–9]	0.14	6 [3–8]	6 [4–8]	0.83
Organ failure (%)	18 (90.0)	29 (72.5)	0.19	9 (75.0)	19 (79.2)	1.0
Shock (%)	5 (25.0)	12 (30.0)	0.78	2 (16.7)	4 (16.7)	1.0
**Outcome**						
ICU length of stay (days) [IQR]	8 [1–11]	5 [2–11]	0.62	4 [3–14]	7 [3–32]	0.40
Death in ICU (%)	2 (10.0)	6 (15.0)	0.71	2 (16.7)	5 (20.8)	1.0
30-day mortality (%)	7 (35.0)	6 (15.0)	0.10	2 (16.7)	6 (25.0)	0.69
90-day mortality (%)	7 (35.0)	12 (30.0)	0.77	4 (33.3)	11 (45.8)	0.72
1-year mortality (%)	8 (40.0)	17 (42.5)	1.0	5 (41.7)	11 (45.8)	1.0

P-values were calculated using fisher’s exact test for comparisons of categorical variables and Mann Whitney U tests or and unpaired t-tests for continuous variables.

Abbreviations: IQR: inter-quartile range, SOFA: sequential organ failure assessment, ICU: intensive care unit

^A^ Including one patient with both central nervous system (CNS) and cardiovascular culture proven infection. This subject was matched to 1 control with CNS infection and 1 with cardiovascular infection.

^B^ This patient was matched to two controls with either skin/soft tissue or abdominal sepsis since these two sites represented the possible sites of infection in the HIV positive patient.

^C^ Without CNS score.

### Impact of HIV co-infection on leukocyte genomic signatures during sepsis

**Fig 1:**
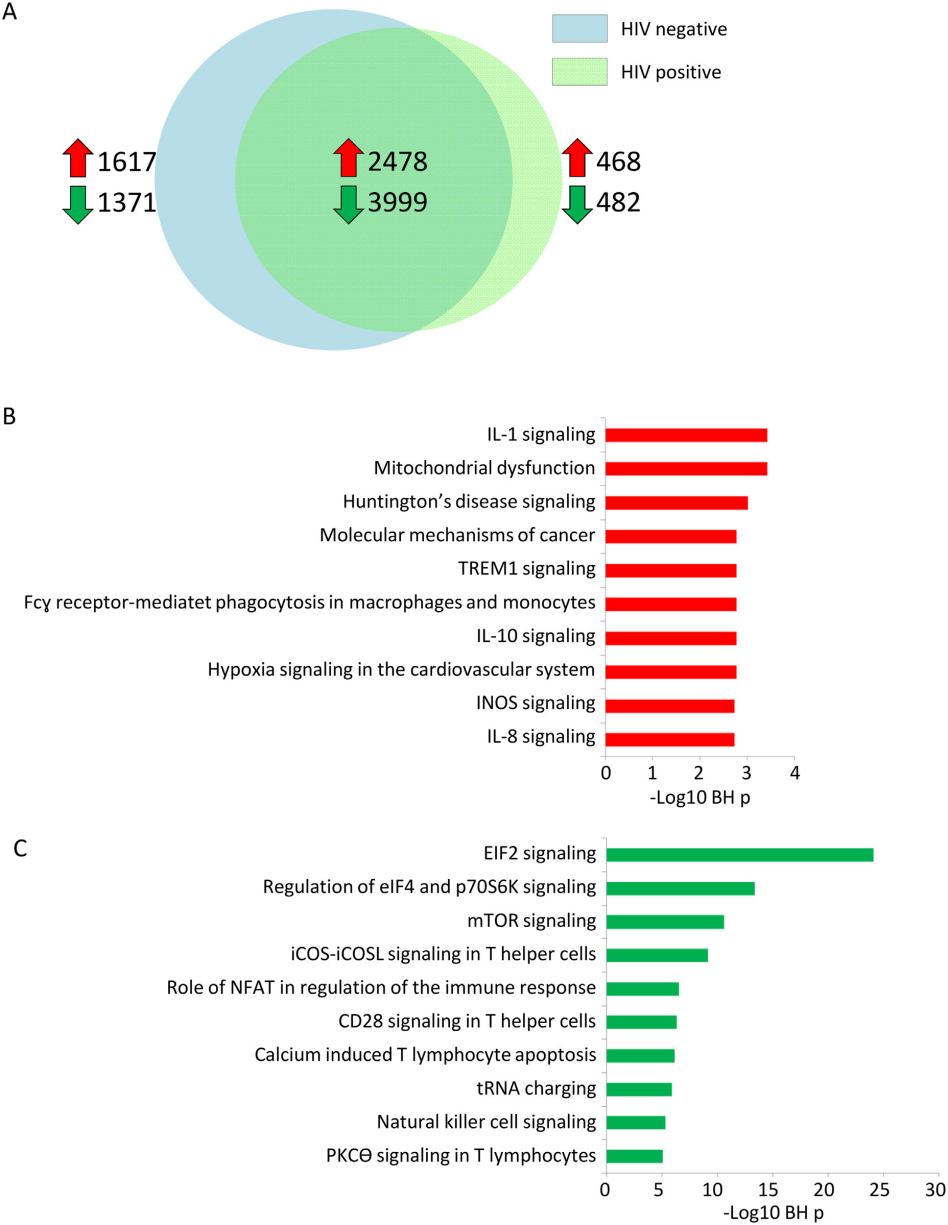
Genomic response to sepsis compared to healthy controls demonstrate a predominantly common host response in sepsis patients with or without HIV infection. (A) Venneuler plot depicting differentially expressed genes between HIV positive and HIV negative sepsis patients as compared to healthy controls. Red and green arrows indicate the number of overexpressed and underexpressed genes, respectively. (B) Top 10 canonical signaling pathways significantly associated with commonly over-expressed genes (n = 2478). Abbreviation:–log (BH) p: negative log10 transformed Benjamini-Hochberg adjusted Fisher’s exact p value. (C) Top 10 canoncical signaling pathways significantly associated with commonly under-expressed genes (n = 3999). Abbreviation:–log (BH) p: negative log10 transformed Benjamini-Hochberg adjusted Fisher’s exact p value.

Whole blood leukocyte gene expression profiles of sepsis patients with or without HIV infection were first compared to healthy controls. Considering BH p<0.05, we observed a predominantly common host response, with 6477 genes significantly altered during sepsis irrespective of HIV status (Fig 1a). Commonly expressed genes were significantly associated with several canonical pathways known to be involved in sepsis pathogenesis, including over expression of IL-1 signaling, IL-10 signaling and TREM-1 signaling (Fig 1b) [27]. Commonly underexpressed pathways included pathways involved in regulation of translation initiation, like EIF2 signaling, and T-helper signaling, such as iCOS-iCOSL signaling and CD28 signaling, pathways which were also uncovered in patients with severe trauma and burn injury (Fig 1c) [28]. Our cohort was not designed to uncover genes associated with mortality and we did not identify significant differences in gene expression profiles between survivors and non-survivors (data not shown).

**Fig 2:**
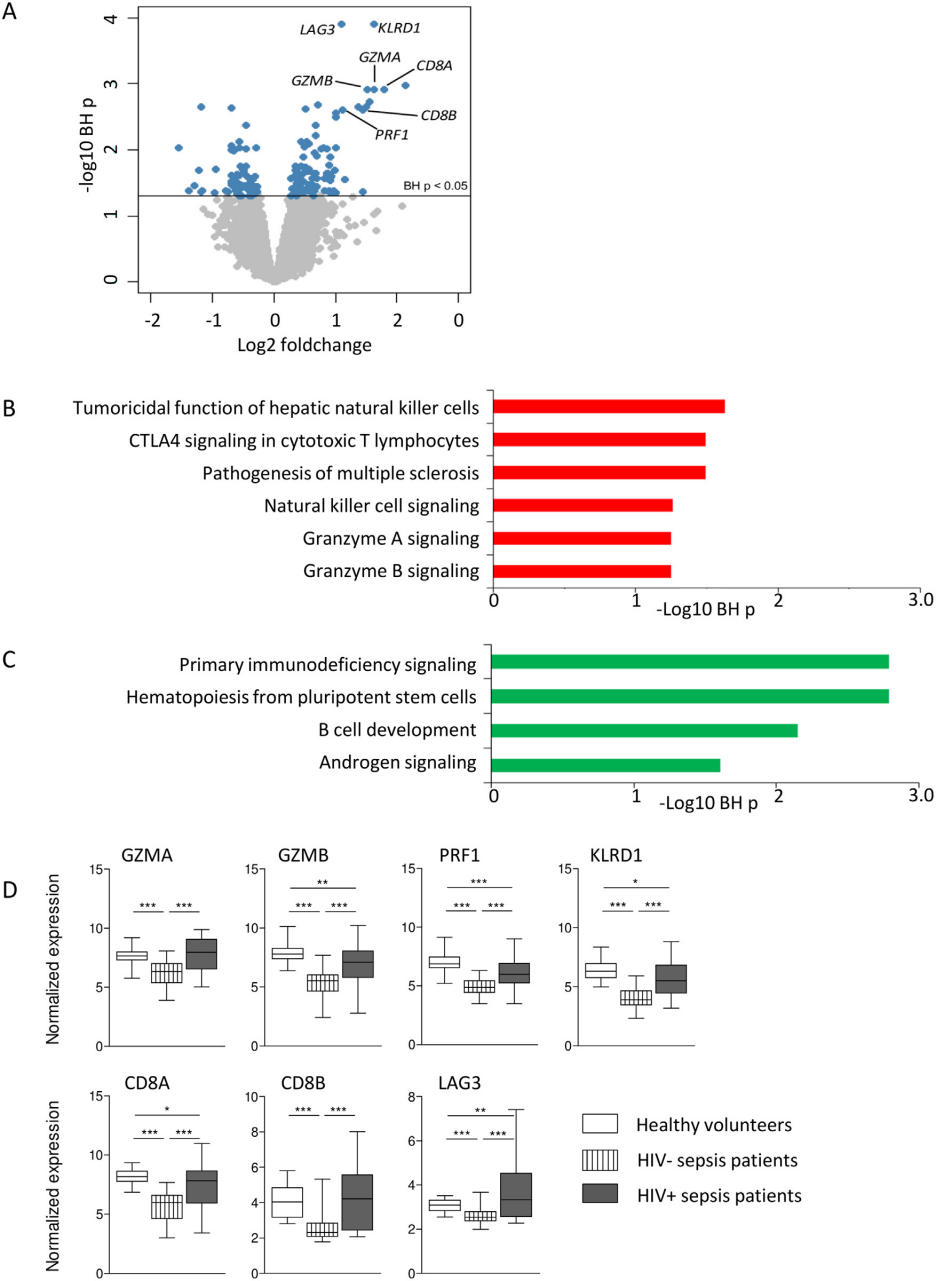
Differential gene expression analysis of HIV positive compared to HIV negative sepsis patient samples in the genomic response cohort reveal differential expression in several canonical pathways. (A) Volcano plot representation (integrating log2 foldchange and multiple comparison adjusted p values) of differential gene expression comparing HIV positive and HIV negative sepsis patients. Horizontal line denotes the multiple comparison adjusted significance threshold (Benjamini-Hochberg (BH) p < 0.05). 149 unique genes were significantly differential (blue dots). (B) Over-expressed genes in HIV positive sepsis patients, as compared to HIV negative sepsis patients, associated to six canonical signaling pathways. Abbreviation:–log (BH) p: negative log10 transformed Benjamini-Hochberg adjusted Fisher’s exact p value. (C) Under-expressed genes in HIV positive sepsis patients, as compared to HIV negative sepsis patients, associated to four canonical signaling pathways. Abbreviation:–log (BH) p: negative log10 transformed Benjamini-Hochberg adjusted Fisher’s exact p value. (D) Expression of prominent genes in pathways differentially expressed between HIV positive and HIV negative sepsis patients. Data are depicted as box- and whisker plots depicting the smallest observation, lower quartile, median, upper quartile and largest observation. * = p<0.05, ** = p<0.01, *** = p<0.001.

**Fig 3:**
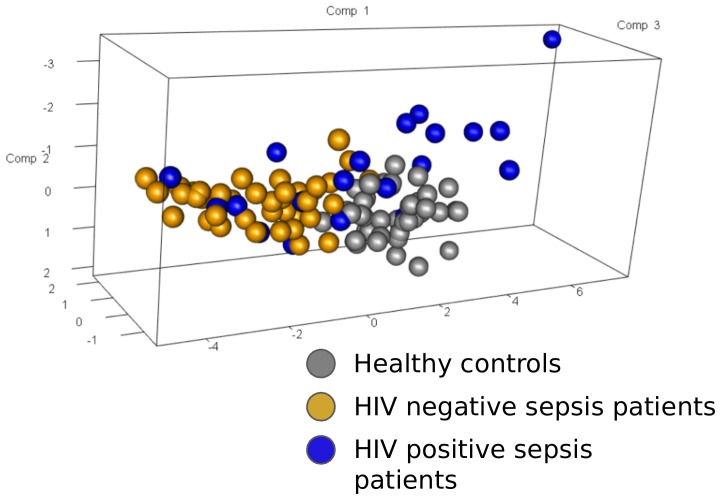
Principal component analysis of *GZMA*, *GZMB*, *CD8A*, *CD8B*, *KLRD1*, *PRF1* and *LAG3* gene expression considering HIV negative and HIV positive sepsis patients. The proportion of explained variance for the first principal component (Comp 1) considering only HIV- and HIV+ patient groups was 81%. Comp2, second principal component; Comp3, third principal component.

Next, we compared the genomic response to sepsis in HIV positive sepsis patients directly to HIV negative sepsis patients. This analysis yielded 149 differentially expressed genes (BH p < 0.05) (Fig 2a). Pathway analysis revealed these to be associated with several canonical pathways (Fig 2b). Over-expressed pathways in HIV positive sepsis patients included those involved in granzyme signaling, natural killer cell signaling and cytotoxic T-cell signaling. Prominent genes in these pathways were granzyme A (*GZMA*), granzyme B (*GZMB*), perforin 1 (*PRF1*), killer cell lectin-like receptor subfamily D, member 1 (*KLRD1*), *CD8A* and *CD8B*. Under-expressed pathways included primary immunodeficiency signaling, hematopoiesis from pluripotent stem cells, B cell development and androgen signaling (Fig 2c). In addition, lymphocyte-activation gene 3 (*LAG3*), involved in inhibitory T-cell signaling was among the most prominent differentially expressed genes. Most of these genes and pathways are remarkably interlinked; granzymes and perforin are released from NK cells and cytotoxic T-cells to promote apoptosis in a target cell [29]. Mature DCs can trigger perforin release and activate KLRD1 on NK cells, resulting in lysis of infected cells, and signal via CD8 to activate cytotoxic T lymphocytes (S1 Fig). LAG3, however, does not play a role in granzyme signaling or cytotoxic T-cell activation, but mediates T-cell suppression by inhibiting the CD3/T-cell reptor complex [30]. Notably, all these prominently differentially expressed genes between HIV positive and HIV negative patients were downregulated in HIV negative sepsis patients when compared to healthy controls. In HIV positive sepsis patients their levels were similar, or downregulated to a lesser extent, except for *LAG3*, which was elevated in HIV positive sepsis patients compared to healthy controls (Fig 2d). Principal component analysis of *GZMA*, *GZMB*, *CD8A*, *CD8B*, *KLRD1*, *PRF1* and *LAG3* gene expression revealed 81% explained variance for the first principal component considering HIV negative and HIV positive sepsis patients (Fig 3).

### Evaluation of differentially expressed genes in an independent cohort of HIV positive and HIV negative patients with sepsis

**Fig 4:**
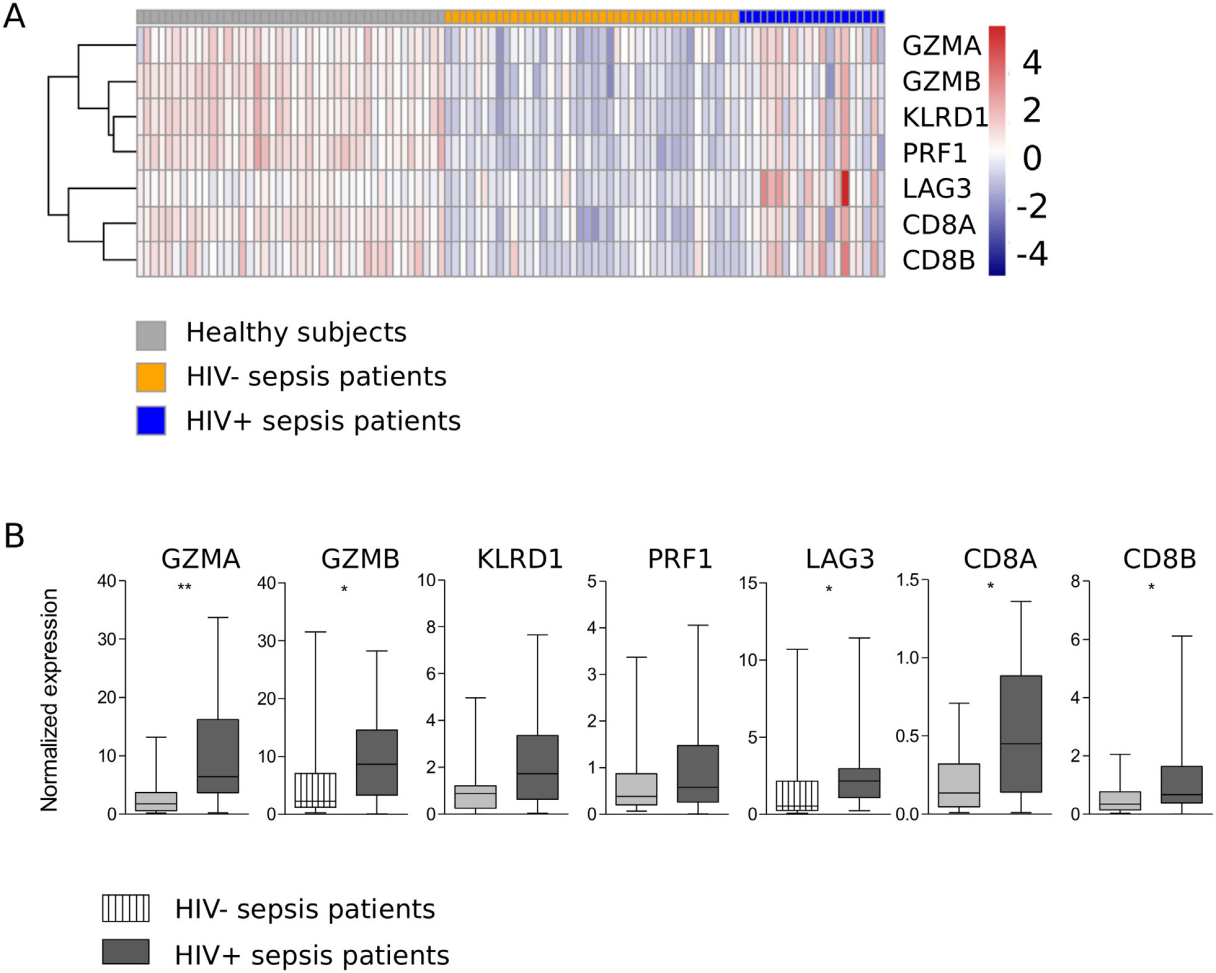
Normalized expression of a validation set of seven genes in an independent cohort of ICU patients with sepsis recruited in a different time frame confirm over-expression in granzyme signaling, cytotoxic T-cell activation genes and T-cell inhibitory genes in HIV positive sepsis patients. (A) Data are depicted as a supervised heatmap representation of significantly differential gene expression indices (BH-adjusted p-value < 0.05) between HIV- and HIV+ patients. Red, over-expressed; blue, under-expressed. (B) Data are also depicted as box- and whisker plots displaying the smallest observation, lower quartile, median, upper quartile and largest observation. * = p<0.05, ** = p<0.01, *** = p<0.001.

On the basis of our blood genomic pathways, we sought to evaluate the expression (qRT-PCR) of genes linked to granzyme signaling, cytotoxic T cell signaling and T cell inhibition, namely *GZMA*, *GZMB*, *KLRD1*, *CD8A*, *CD8B*, *LAG3*, *PRF1* and *KLRD1* (Fig 4) in a second independent cohort of ICU patients consisting of 12 HIV positive and 24 matched HIV negative patients with sepsis. Demographics of these patients were comparable to the genomic response cohort (Table 1). The respiratory tract was again the most common site of infection and *Pneumocystis jirovecii* and *Candida albicans* were exclusively found in HIV positive patients (S2 Table). Disease severity and mortality did not differ between HIV positive and HIV negative sepsis patients (Table 1) and there were no differences in viral loads or CD4 counts between survivors and non-survivors at any time point. In this independent cohort, we confirmed differential regulation of *GZMA*, *GZMB*, *CD8A*, *CD8B* and *LAG3*, but not *PRF1* and *KLRD1* (Fig 4). These results support the robustness of over-expression in granzyme signaling, cytotoxic T-cell activation genes and T-cell inhibitory genes in HIV positive sepsis patients compared to HIV negative sepsis patients upon ICU admission. *PRF1* was significantly lower in non-survivors (at 30 days after ICU admission) in patients with HIV co-infection (p = 0.03), but there were no other genes associated with mortality. However, the numbers of non-survivors were small, specifically in the HIV positive group (n = 2).

### Impact of asymptomatic HIV infection on the expression of genes of interest

**Fig 5:**
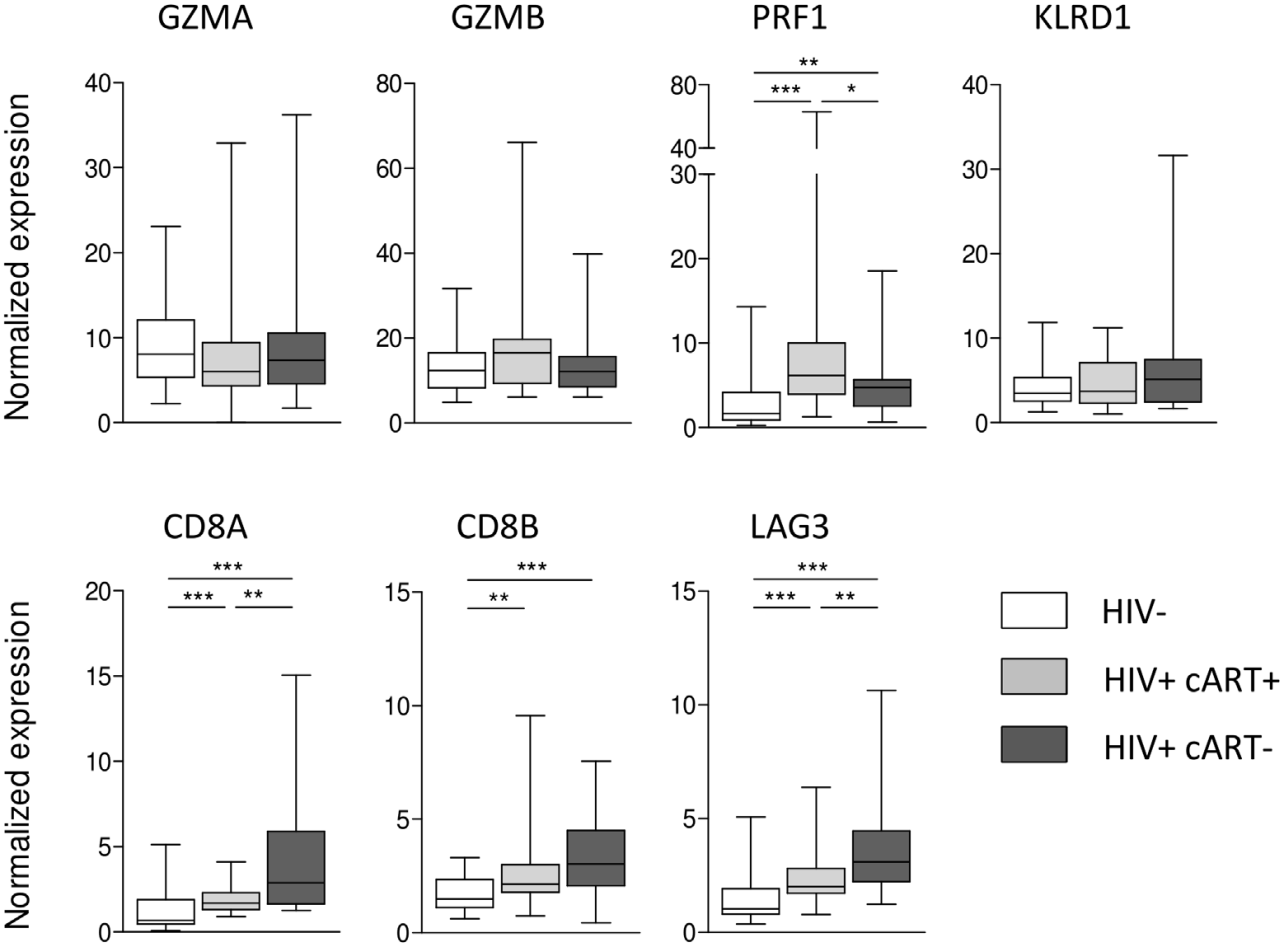
Normalized expression of a validation set of seven genes in asymptomatic subjects demonstrate overexpression of cytotoxic T-cell signaling (*CD8A*, *CD8B*) and T-cell inhibitory signaling in HIV patients. Data are depicted as box- and whisker plots depicting the smallest observation, lower quartile, median, upper quartile and largest observation. * = p<0.05, ** = p<0.01, *** = p<0.001.

Differential gene expression in HIV positive and HIV negative sepsis patients could be driven by HIV infection per se, or become apparent during severe bacterial infection. Therefore, we next determined the influence of HIV infection per se and the impact of cART on expression of genes with enhanced expression in HIV positive sepsis patients. We used a cohort of 60 asymptomatic HIV positive patients and 33 healthy controls without HIV infection (S3 Table). In this population, mRNA’s encoding *PRF1*, *CD8A*, *CD8B*, and *LAG3* were upregulated in HIV infected individuals; while *CD8A*, *CD8B*, and *LAG3* expression especially was high in HIV patients not using cART. *PRF1* expression was highest in HIV patients on cART. HIV infection did not influence the expression of *GZMA*, *GZMB* or *KLRD1* (Fig 5). No correlations were observed between these genes and CD4 counts, but CD8A and LAG3 correlated with CD8 counts (r = 0.48, p = 0.0002 and r = 0.35, p = 0.01, respectively) and CD8B and LAG3 correlated with viral load (r = 0.31, p = 0.018 and r = 0.36, p = 0.006, respectively) (S4 Table).

### Plasma levels of granzymes

Finally, we assessed whether HIV co-infection also impacts on plasma levels of circulating granzyme A and granzyme B in ICU patients with sepsis. Samples were available for 30 HIV positive patients and 39 HIV negative patients. For both granzyme A and granzyme B, a large number of samples was below the detection limit (81.3% and 36.1%, for granzyme A and granzyme B respectively), and no differences were observed according to HIV status (S2 Fig).

## Discussion

We examined the impact of HIV infection on host leukocyte responses in sepsis patients. Applying blood gene expression profiling and pathway analyses in a cohort of septic ICU patients with or without HIV infection we revealed a predominantly common host response. However, we also identified an HIV specific signature of genes involved in granzyme signaling (*GZMA*, *GZMB*, *PRF1*), natural killer cell signaling (*KLRD1*), cytotoxic T-cell signaling (*CD8A*, *CD8B*) and T-cell inhibitory signaling (*LAG3*). The robustness of our findings was confirmed for *GZMA*, *GZMB*, *CD8A*, *CD8B* and *LAG3* by qRT-PCR analysis in an independent validation cohort independent of the genomic response cohort. Importantly, enhanced expression of *PRF1*, *CD8A*, *CD8B* and *LAG3* was also unmasked in a cohort of asymptomatic HIV patients.

Previous studies reported that HIV impacts on several aspects of the immune system involved in sepsis pathogenesis[6]. In this respect, it is interesting that we found a predominantly common host response to sepsis in HIV positive and HIV negative patients. On the other hand, previous studies have shown a high level of similarity on a transcriptome level between patients with sepsis and other causes of systemic inflammation [31], and between patients with sepsis caused by different pathogens [32, 33]. Indeed, there is mounting evidence for a “common host response” that is expressed by several different cell types in response to a diverse range of pathogens [33]. Prominent genes within the common host response include genes that mediate cytokine and chemokine production, interferon-stimulated genes, genes that activate the immune response, genes that limit the immune response, and genes involved in lymphocyte activation, antigen presentation, cell adhesion and tissue invasion [33]. In addition, the host response to HIV infection overlaps with genes in the common host response to a variety of infections. For instance, HIV infection activates interferon-stimulated genes [33], and monocytes from chronically HIV-1 infected subjects expressed a gene activation signature similar to monocytes from uninfected subjects following de novo stimulation with a TLR2 agonist [34]. These similarities in the host response to HIV and sepsis could result in overlapping gene expression profiles in sepsis patients with HIV co-infection, and thus a high level of uniformity with HIV negative sepsis patients.

Cytotoxic T-cell signaling (*CD8A*, *CD8B*) and T-cell inhibitory signaling (*LAG3*) were overexpressed in HIV patients both during sepsis and in asymptomatic HIV infection. These findings indicate an effect of HIV infection per se, which persists during sepsis. In HIV negative sepsis patients, a reduction in *CD8A* and *CD8B* is consistent with immune suppression, which is considered an integral part of the host response to sepsis, and also involves enhanced apoptosis of CD8 T cells [35]. In HIV positive patients, overexpression of *CD8A* and *CD8B* could be related to increased numbers of CD8 T-cells. CD8 T-cell expansion is commonly observed in early HIV infection and persists in some patients [36]. Our finding of increased LAG3 expression in asymptomatic HIV patients is in accordance with the literature. Previous studies found LAG3 to be upregulated in T-cells of patients with HIV and LAG3 is considered a marker of ‘immune exhaustion’ [37, 30], characterized by functional unresponsiveness of effector or activated T-cells [37]. Blocking of LAG3 results in T-cell proliferation and release of TH1 cytokines [37]. Hence, downregulation of LAG3 in HIV negative sepsis patients may be an adequate response to boost the immune system. Hypothetically, increased expression of LAG3 could be a disadvantage in HIV positive sepsis patients by inhibiting an effective T-cell response. However, in this study we observed neither differences in mortality rates according to HIV status, nor did LAG3 correlate with survival. Therefore, the clinical relevance of LAG3 overexpression during sepsis in HIV positive patients appears limited.

*GZMA* and *GZMB* were under-expressed in sepsis patients without HIV infection, but not in HIV positive sepsis patients, and differences in granzyme signaling were not apparent in asymptomatic HIV patients. Plasma levels of granzymes were largely below the detection limit of our assay, which is in accordance with a previous study on granzymes in sepsis patients [38]. As granzyme signaling is a predominantly intercellular process [39], plasma levels may not be representative of granzyme activity.

This study is limited by the fact that examination of the host response on a gene expression level does not allow for firm conclusions on protein expression and the physiological consequences thereof. Furthermore, as the number of HIV patients was limited, we were unable to stratify patients according to HIV disease progression and immune suppression, and our numbers were insufficient to reliably assess associations of differentially expressed genes with mortality. However, our method did allow for the assessment of a wide range of genes, which is unprecedented in HIV co-infected sepsis patients.

## Conclusion

We demonstrated that, on a gene expression level, sepsis results in a predominantly common response in patients with or without HIV co-infection. Several genes, involved in granzyme signaling, cytotoxic T-cell signaling and T-cell inhibitory signaling, were differentially expressed according to HIV status. Our findings suggest that these differences were largely related to changes commonly observed in asymptomatic HIV patients which persist during sepsis.

## Supporting Information

S1 Fig Ingenuity canonical signaling pathways associated with over-expressed genes in HIV positive sepsis patients as compared to HIV negative sepsis patients. (Figure A) granzyme A signaling, and (Figure B) CTLA4 signaling in cytotoxic T lymphocytes. Red colored genes denote over-expression in HIV positive sepsis patients.
(PDF)

S2 Fig Plasma levels of granzyme A and granzyme B in sepsis patients with or without HIV infection. (PDF)

S1 Table Primer sequences for qPCR analysis. (DOC)

S2 Table Causative pathogens of sepsis in the genomic response cohort and validation cohort. (DOC)

S3 Table Characteristics of asymptomatic subjects with or without HIV infection from a HIV endemic region. (DOC)

S4 Table Correlations between genes of interest and CD4 count or viral load in asymptomatic HIV patients. (DOC)

## References

[1] Rosenberg AL, Seneff MG, Atiyeh L, Wagner R, Bojanowski L, Zimmerman JE. The importance of bacterial sepsis in intensive care unit patients with acquired immunodeficiency syndrome: implications for future care in the age of increasing antiretroviral resistance. Crit Care Med. 2001;29(3):548–56. 11373418 10.1097/00003246-200103000-00013

[2] Chiang HH, Hung CC, Lee CM, Chen HY, Chen MY, Sheng WH et al Admissions to intensive care unit of HIV-infected patients in the era of highly active antiretroviral therapy: etiology and prognostic factors. Crit Care. 2011;15(4):R202 10.1186/cc10419 21871086 PMC3387644

[3] Huson MA, Stolp SM, van der Poll T, Grobusch MP. Community-acquired bacterial bloodstream infections in HIV-infected patients: a systematic review. Clin Infect Dis. 2014;58(1):79–92. 10.1093/cid/cit596 24046307

[4] Levy MM, Fink MP, Marshall JC, Abraham E, Angus D, Cook D et al 2001 SCCM/ESICM/ACCP/ATS/SIS International Sepsis Definitions Conference. Crit Care Med. 2003;31(4):1250–6. 10.1097/01.CCM.0000050454.01978.3B 12682500

[5] Angus DC, van der Poll T. Severe sepsis and septic shock. N Engl J Med. 2013;369(9):840–51. 10.1056/NEJMra1208623 23984731

[6] Huson MA, Grobusch MP, van der Poll T. The effect of HIV infection on the host response to bacterial sepsis. Lancet Infect Dis. 2015;15(1):95–108. 10.1016/S1473-3099(14)70917-X 25459220

[7] Silva JM Jr., dos Santos S de S. Sepsis in AIDS patients: clinical, etiological and inflammatory characteristics. J Int AIDS Soc. 2013;16:17344 10.7448/IAS.16.1.17344 23374857 PMC3564973

[8] Amancio RT, Japiassu AM, Gomes RN, Mesquita EC, Assis EF, Medeiros DM et al The innate immune response in HIV/AIDS septic shock patients: a comparative study. PLoS One. 2013;8(7):e68730 10.1371/journal.pone.0068730 23874739 PMC3708901

[9] Mankhambo LA, Banda DL, Group IPDS, Jeffers G, White SA, Balmer P et al The role of angiogenic factors in predicting clinical outcome in severe bacterial infection in Malawian children. Crit Care. 2010;14(3):R91 10.1186/cc9025 20492647 PMC2911728

[10] Huson MA, Wouters D, van Mierlo G, Grobusch MP, Zeerleder SS, van der Poll T. HIV Coinfection Enhances Complement Activation During Sepsis. J Infect Dis. 2015 10.1093/infdis/jiv074 25657259

[11] Wong HR. Clinical review: sepsis and septic shock—the potential of gene arrays. Crit Care. 2012;16(1):204 10.1186/cc10537 22316118 PMC3396217

[12] Tang BM, Huang SJ, McLean AS. Genome-wide transcription profiling of human sepsis: a systematic review. Crit Care. 2010;14(6):R237 10.1186/cc9392 21190579 PMC3219990

[13] Klein Klouwenberg PM, Ong DS, Bos LD, de Beer FM, van Hooijdonk RT, Huson MA et al Interobserver agreement of Centers for Disease Control and Prevention criteria for classifying infections in critically ill patients. Crit Care Med. 2013;41(10):2373–8. 10.1097/CCM.0b013e3182923712 23921277

[14] Vincent JL, Moreno R, Takala J, Willatts S, De Mendonca A, Bruining H et al The SOFA (Sepsis-related Organ Failure Assessment) score to describe organ dysfunction/failure. On behalf of the Working Group on Sepsis-Related Problems of the European Society of Intensive Care Medicine. Intensive Care Med. 1996;22(7):707–10. 8844239 10.1007/BF01709751

[15] Kaukonen KM, Bailey M, Suzuki S, Pilcher D, Bellomo R. Mortality related to severe sepsis and septic shock among critically ill patients in Australia and New Zealand, 2000–2012. JAMA. 2014;311(13):1308–16. 10.1001/jama.2014.2637 24638143

[16] Garner JS, Jarvis WR, Emori TG, Horan TC, Hughes JM. CDC definitions for nosocomial infections, 1988. Am J Infect Control. 1988;16(3):128–40. 2841893 10.1016/0196-6553(88)90053-3

[17] Calandra T, Cohen J, International Sepsis Forum Definition of Infection in the ICUCC. The international sepsis forum consensus conference on definitions of infection in the intensive care unit. Crit Care Med. 2005;33(7):1538–48. 16003060 10.1097/01.ccm.0000168253.91200.83

[18] Levy MM, Fink MP, Marshall JC, Abraham E, Angus D, Cook D et al 2001 SCCM/ESICM/ACCP/ATS/SIS International Sepsis Definitions Conference. Intensive Care Med. 2003;29(4):530–8. 10.1007/s00134-003-1662-x 12664219

[19] Gautier L, Cope L, Bolstad BM, Irizarry RA. affy—analysis of Affymetrix GeneChip data at the probe level. Bioinformatics. 2004;20(3):307–15. 10.1093/bioinformatics/btg405 14960456

[20] Bourgon R, Gentleman R, Huber W. Independent filtering increases detection power for high-throughput experiments. Proc Natl Acad Sci U S A. 2010;107(21):9546–51. 10.1073/pnas.0914005107 20460310 PMC2906865

[21] Leek JT, Storey JD. Capturing heterogeneity in gene expression studies by surrogate variable analysis. PLoS Genet. 2007;3(9):1724–35. 10.1371/journal.pgen.0030161 17907809 PMC1994707

[22] Johnson WE, Li C, Rabinovic A. Adjusting batch effects in microarray expression data using empirical Bayes methods. Biostatistics. 2007;8(1):118–27. 10.1093/biostatistics/kxj037 16632515

[23] Smyth G. Limma: linear models for microarray data Bioinformatics and Computational Biology Solutions using R and Bioconductor. New York: Springer; 2005 p. 397–420.

[24] van Lieshout MH, Scicluna BP, Florquin S, van der Poll T. NLRP3 and ASC differentially affect the lung transcriptome during pneumococcal pneumonia. Am J Respir Cell Mol Biol. 2014;50(4):699–712. 10.1165/rcmb.2013-0015OC 24164497

[25] Ruijter JM, Pfaffl MW, Zhao S, Spiess AN, Boggy G, Blom J et al Evaluation of qPCR curve analysis methods for reliable biomarker discovery: bias, resolution, precision, and implications. Methods. 2013;59(1):32–46. 10.1016/j.ymeth.2012.08.011 22975077

[26] Spaeny-Dekking EH, Hanna WL, Wolbink AM, Wever PC, Kummer JA, Swaak AJ et al Extracellular granzymes A and B in humans: detection of native species during CTL responses in vitro and in vivo. J Immunol. 1998;160(7):3610–6. 9531325

[27] Parlato M, Cavaillon JM. Host response biomarkers in the diagnosis of sepsis: a general overview. Methods Mol Biol. 2015;1237:149–211. 10.1007/978-1-4939-1776-1_15 25319788

[28] Xiao W, Mindrinos MN, Seok J, Cuschieri J, Cuenca AG, Gao H et al A genomic storm in critically injured humans. J Exp Med. 2011;208(13):2581–90. 10.1084/jem.20111354 22110166 PMC3244029

[29] Ewen CL, Kane KP, Bleackley RC. A quarter century of granzymes. Cell Death Differ. 2012;19(1):28–35. 10.1038/cdd.2011.153 22052191 PMC3252830

[30] Larsson M, Shankar EM, Che KF, Saeidi A, Ellegard R, Barathan M et al Molecular signatures of T-cell inhibition in HIV-1 infection. Retrovirology. 2013;10:31 10.1186/1742-4690-10-31 23514593 PMC3610157

[31] Scicluna B, van Vught LA, Klein Klouwenberg PM, Wiewel MA, Ong DS, Zwinderman AH et al A molecular biomarker to diagnose community-acquired pneumonia on intensive care unit admission. Am J Respir Crit Care Med. 2015;192(7):826–35. 10.1164/rccm.201502-0355OC 26121490

[32] Tang BM, McLean AS, Dawes IW, Huang SJ, Cowley MJ, Lin RC. Gene-expression profiling of gram-positive and gram-negative sepsis in critically ill patients. Crit Care Med. 2008;36(4):1125–8. 10.1097/CCM.0b013e3181692c0b 18379237

[33] Jenner RG, Young RA. Insights into host responses against pathogens from transcriptional profiling. Nat Rev Microbiol. 2005;3(4):281–94. 10.1038/nrmicro1126 15806094

[34] Gekonge B, Giri MS, Kossenkov AV, Nebozyhn M, Yousef M, Mounzer K et al Constitutive gene expression in monocytes from chronic HIV-1 infection overlaps with acute Toll-like receptor induced monocyte activation profiles. PLoS One. 2012;7(7):e41153 10.1371/journal.pone.0041153 22815948 PMC3399809

[35] Hotchkiss RS, Monneret G, Payen D. Immunosuppression in sepsis: a novel understanding of the disorder and a new therapeutic approach. Lancet Infect Dis. 2013;13(3):260–8. 10.1016/S1473-3099(13)70001-X 23427891 PMC3798159

[36] Smith PR, Cavenagh JD, Milne T, Howe D, Wilkes SJ, Sinnott P et al Benign monoclonal expansion of CD8+ lymphocytes in HIV infection. J Clin Pathol. 2000;53(3):177–81. 10823134 10.1136/jcp.53.3.177 PMC1731162

[37] Khaitan A, Unutmaz D. Revisiting immune exhaustion during HIV infection. Curr HIV/AIDS Rep. 2011;8(1):4–11. 10.1007/s11904-010-0066-0 21188556 PMC3144861

[38] Zeerleder S, Hack CE, Caliezi C, van Mierlo G, Eerenberg-Belmer A, Wolbink A et al Activated cytotoxic T cells and NK cells in severe sepsis and septic shock and their role in multiple organ dysfunction. Clin Immunol. 2005;116(2):158–65. 10.1016/j.clim.2005.03.006 15993363

[39] Lieberman J. The ABCs of granule-mediated cytotoxicity: new weapons in the arsenal. Nat Rev Immunol. 2003;3(5):361–70. 10.1038/nri1083 12766758

