# Supplementary material for: The Impact of HIV Co-Infection on the Genomic Response to Sepsis
Source: PLoS One. 2016 Feb 12;11(2):e0148955. doi: 10.1371/journal.pone.0148955 (PMC4752296; doi:10.1371/journal.pone.0148955)

S1 Figure: Ingenuity canonical signaling pathways associated with over-expressed genes in HIV positive sepsis patients as compared to HIV negative sepsis patients. (A) granzyme A signaling, and (B) CTLA4 signaling in cytotoxic T lymphocytes. Red colored genes denote over-expression in HIV positive sepsis patients.

A

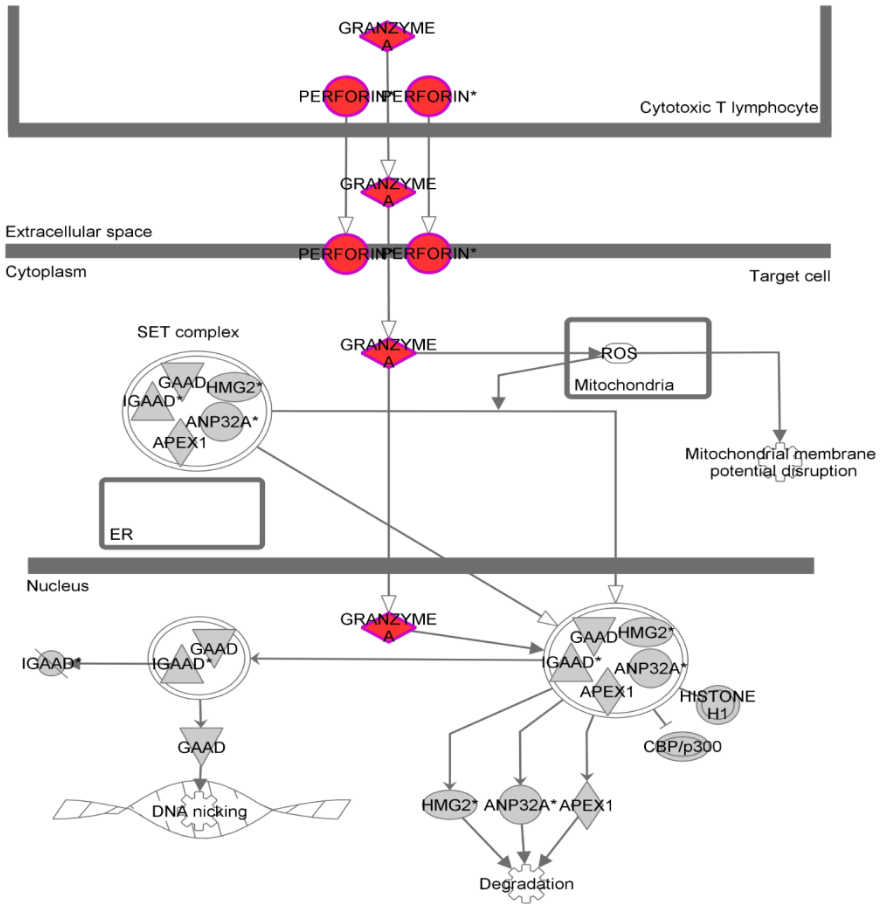

B

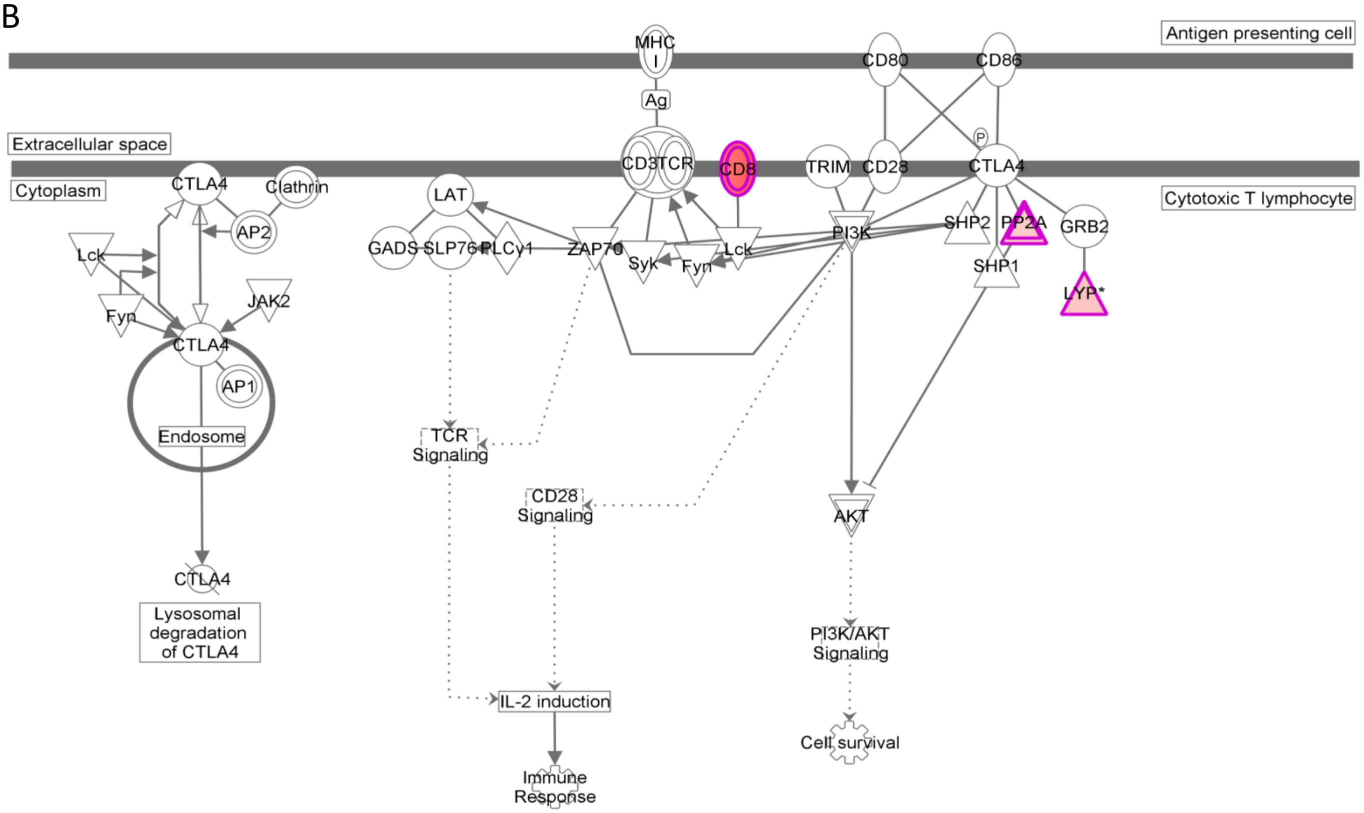

Supplement: S1 Fig — (Figure A) granzyme A signaling, and (Figure B) CTLA4 signaling in cytotoxic T lymphocytes. Red colored genes denote over-expression in HIV positive sepsis patients. (PDF) [file pone.0148955.s001.pdf]
