# Supplementary material for: The Impact of HIV Co-Infection on the Genomic Response to Sepsis
Source: PLoS One. 2016 Feb 12;11(2):e0148955. doi: 10.1371/journal.pone.0148955 (PMC4752296; doi:10.1371/journal.pone.0148955)
Supplement: S2 Fig — (PDF) [file pone.0148955.s002.pdf]

S2 Figure: Plasma levels of granzyme A and granzyme B in sepsis patients with or without HIV infection.

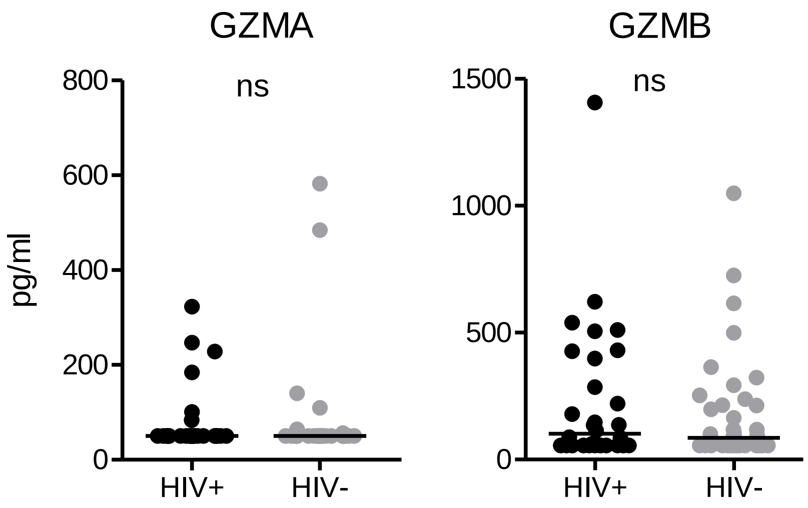

The limit of detection was 50 pg/ml for granzyme A and 55 pg/ml for granzyme B.
Horizontal lines indicate medians.
Abbreviations: GZMA: granzyme A. GZMB: granzyme B
