# Supplementary material for: The Impact of HIV Co-Infection on the Genomic Response to Sepsis
Source: PLoS One. 2016 Feb 12;11(2):e0148955. doi: 10.1371/journal.pone.0148955 (PMC4752296; doi:10.1371/journal.pone.0148955)
Supplement: S1 Table — (DOC) [file pone.0148955.s003.doc]

**S1 Table**: Primer sequences for qPCR analysis

| **Gene** | **Forward primer** | **Reverse primer** |
| --- | --- | --- |
| *HPRT1* | 5’ GGATTTGAAATTCCAGACAAGTTT | 5’ GCGATGTCAATAGGACTCCAG |
| *GZMA* | 5’ TATGGTTTGTGCTGGAAGCC | 5’ TATAGACACCAGGCCCACGAG |
| *GZMB* | 5’ GATCGAAAGTGCGAATCTGAC | 5’ GCCATTGTTTCGTCCATAGG |
| *KLRD1* | 5’ GCCTGCTTCAGCTTCAAAAC | 5’ AGCCATTCTCCCACAACCAG |
| *PRF1* | 5’ AACTTTGCAGCCCAGAAGAC | 5’ CTCTTGAAGTCAGGGTGCAG |
| *LAG3* | 5’ CATATCCATCTGCAGGAACAGC | 5’ GCTCCACACAAAGCGTTCTTG |
| *CD8A* | 5’ GCTGGACTTCGCCTGTGATA | 5’ AAACACGTCTTCGGTTCCTG |
| *CD8B* | 5’ TTTGTAGCCCCATCACCCTTG | 5’ AAACGAAGCCGGGCTCTC |
