# Supplementary material for: The Impact of HIV Co-Infection on the Genomic Response to Sepsis
Source: PLoS One. 2016 Feb 12;11(2):e0148955. doi: 10.1371/journal.pone.0148955 (PMC4752296; doi:10.1371/journal.pone.0148955)
Supplement: S2 Table — (DOC) [file pone.0148955.s004.doc]

**S2 Table: Causative pathogens of sepsis in the genomic response cohort and validation cohort.**

|  | **Genomic response cohort** | | |  | **Validation cohort** | | |
| --- | --- | --- | --- | --- | --- | --- | --- |
|  | HIV+  n=20 | HIV-  n=40 | p-value |  | HIV+ n=12 | HIV- n=24 | p-value |
| *Staphylococcus aureus* | 5 (25.0) | 16 (40.0) | 0.39 |  | 0 (0.0) | 6 (25.0) | 0.08 |
| *Streptococcus pneumoniae* | 3 (15.0) | 4 (10.0) | 0.67 |  | 1 (8.3) | 3 (12.5) | 1.0 |
| *Enterococcus faecium* | 2 (10.0) | 8 (20.0) | 0.47 |  | 0 (0.0) | 0 (0.0) | 1.0 |
| *Escherichia coli* | 3 (15.0) | 6 (15.0) | 1.0 |  | 0 (0) | 1 (4.2) | 1.0 |
| *Pseudomonas aeruginosa* | 0 (0.0) | 7 (17.5) | 0.08 |  | 0 (0) | 2 (8.3) | 0.54 |
| *Staphylococcus epidermidis* (CNS) | 1 (5.0) | 3 (7.5) | 1.0 |  | 2 (16.7) | 2 (8.3) | 0.59 |
| *Pneumocystis jirovecii* | 3 (15.0) | 0 (0.0) | **0.03** |  | 4 (33.3) | 0 (0) | **0.008** |
| Influenza virus (incl. H1N1) | 0 (0.0) | 2 (5.0) | 0.55 |  | 0 (0) | 3 (12.5) | 0.55 |
| *Serratia marcescens* | 0 (0.0) | 4 (10.0) | 0.29 |  | 0 (0) | 2 (8.3) | 0.54 |
| *Candida albicans* | 1 (5.0) | 0 (0.0) | 0.33 |  | 3 (25.0) | 0 (0) | **0.03** |
| *Citrobacter species* | 0 (0.0) | 3 (7.5) | 0.54 |  | 0 (0) | 1 (4.2) | 1.0 |
| *Cytomegalovirus* | 1 (5.0) | 0 (0.0) | 0.33 |  | 2 (16.7) | 1 (4.2) | 0.25 |
| *Haemophilus influenzae* | 0 (0.0) | 1 (2.5) | 1.0 |  | 0 (0) | 3 (12.5) | 0.55 |
| *Streptococcus species* | 0 (0.0) | 3 (7.5) | 0.54 |  | 0 (0) | 1 (4.2) | 1.0 |
