# Supplementary material for: The Impact of HIV Co-Infection on the Genomic Response to Sepsis
Source: PLoS One. 2016 Feb 12;11(2):e0148955. doi: 10.1371/journal.pone.0148955 (PMC4752296; doi:10.1371/journal.pone.0148955)
Supplement: S3 Table — (DOC) [file pone.0148955.s005.doc]

**S3 Table: Characteristics of asymptomatic subjects with or without HIV infection from a HIV endemic region.**

|  | HIV-  n=33 | HIV+ on cART  n=34 | HIV+ non cART  n=26 | P |
| --- | --- | --- | --- | --- |
| **Demographics** |  |  |  |  |
| Age (years) | 38 (27-43) | 46 (36-53) | 37 (32-48) | 0.11 |
| Males | 16 (48.5) | 12 (35.3) | 8 (30.8) | 0.35 |
| **HIV disease progression** |  |  |  |  |
| CD4 count (cells/mm^3^) | -- | 302 (189-480) | 460 (320-642) | 0.038 |
| Viral load (cp/ml)^A^ | -- | 200 (200-200) | 1.26 x10^5^ (200-6.91 x10^5^) | 0.0001 |

Continuous variables are presented as medians with the interquartile range. Categorical variables are presented as a number and percentage. P-values were calculated using X^2^ tests for categorical variables and one-way ANOVA, unpaired t-tests or Mann-Whitney U tests as appropriate for continuous variables.
Abbreviations: cART: combination antiretroviral therapy; cp: copies.
^A^ The lower limit of detection was 200 cp/ml. Patients with values below 200 were set at 200 cp/ml.
