# Supplementary material for: The Impact of HIV Co-Infection on the Genomic Response to Sepsis
Source: PLoS One. 2016 Feb 12;11(2):e0148955. doi: 10.1371/journal.pone.0148955 (PMC4752296; doi:10.1371/journal.pone.0148955)
Supplement: S4 Table — (DOC) [file pone.0148955.s006.doc]

**S4 Table: Correlations between genes of interest and CD4 count or viral load in asymptomatic HIV patients.**

|  |  | **CD4** | |  | **CD8** | |  | **Viral load** | |
| --- | --- | --- | --- | --- | --- | --- | --- | --- | --- |
|  |  | *R* | *P* |  | *R* | *P* |  | *R* | *P* |
| GZMA |  | -0.22 | 0.09 |  | 0.05 | 0.73 |  | -0.11 | 0.41 |
| GZMB |  | 0.00 | 0.97 |  | 0.08 | 0.58 |  | -0.23 | 0.08 |
| PRF1 |  | -0.19 | 0.16 |  | -0.02 | 0.91 |  | -0.08 | 0.54 |
| KLRD1 |  | -0.18 | 0.17 |  | 0.16 | 0.25 |  | -0.06 | 0.67 |
| CD8A |  | 0.09 | 0.50 |  | **0.48** | **0.0002** |  | 0.18 | 0.17 |
| CD8B |  | -0.07 | 0.58 |  | 0.16 | 0.25 |  | **0.31** | **0.02** |
| LAG3 |  | 0.00 | 1.0 |  | **0.35** | **0.01** |  | **0.36** | **0.006** |

Spearman rank tests were used to examine correlations.
